# Macrophagic and microglial responses after focal traumatic brain injury in the female rat

**DOI:** 10.1186/1742-2094-11-82

**Published:** 2014-04-24

**Authors:** L Christine Turtzo, Jacob Lescher, Lindsay Janes, Dana D Dean, Matthew D Budde, Joseph A Frank

**Affiliations:** 1 Center for Neuroscience and Regenerative Medicine, Uniformed Services University of the Health Sciences, 4301 Jones Bridge Road, Bethesda, MD 20814, USA; 2 Frank Laboratory, National Institutes of Health, Building 10, Room B1N256, MSC 1074, 10 Center Drive, Bethesda, MD 20814, USA; 3 National Institute of Biomedical Imaging and Bioengineering, National Institutes of Health, 10 Center Drive, Bethesda, MD 20814, USA; 4 Department of Environmental Science, Baylor University, One Bear Place #97266, Waco, TX 76798, USA; 5 Department of Neurosurgery, Medical College of Wisconsin, 8701 Watertown Plank Road, Milwaukee, WI 53226, USA

**Keywords:** Controlled cortical impact, Inflammation, Macrophage, Microglia, MRI, Rat, Traumatic brain injury

## Abstract

**Background:**

After central nervous system injury, inflammatory macrophages (M1) predominate over anti-inflammatory macrophages (M2). The temporal profile of M1/M2 phenotypes in macrophages and microglia after traumatic brain injury (TBI) in rats is unknown. We subjected female rats to severe controlled cortical impact (CCI) and examined the postinjury M1/M2 time course in their brains.

**Methods:**

The motor cortex (2.5 mm left laterally and 1.0 mm anteriorly from the bregma) of anesthetized female Wistar rats (ages 8 to 10 weeks; *N* = 72) underwent histologically moderate to severe CCI with a 5-mm impactor tip. Separate cohorts of rats had their brains dissociated into cells for flow cytometry, perfusion-fixed for immunohistochemistry (IHC) and *ex vivo* magnetic resonance imaging or flash-frozen for RNA and protein analysis. For each analytical method used, separate postinjury times were included for 24 hours; 3 or 5 days; or 1, 2, 4 or 8 weeks.

**Results:**

By IHC, we found that the macrophagic and microglial responses peaked at 5 to 7 days post-TBI with characteristics of mixed populations of M1 and M2 phenotypes. Upon flow cytometry examination of immunological cells isolated from brain tissue, we observed that peak M2-associated staining occurred at 5 days post-TBI. Chemokine analysis by multiplex assay showed statistically significant increases in macrophage inflammatory protein 1α and keratinocyte chemoattractant/growth-related oncogene on the ipsilateral side within the first 24 hours after injury relative to controls and to the contralateral side. Quantitative RT-PCR analysis demonstrated expression of both M1- and M2-associated markers, which peaked at 5 days post-TBI.

**Conclusions:**

The responses of macrophagic and microglial cells to histologically severe CCI in the female rat are maximal between days 3 and 7 postinjury. The response to injury is a mixture of M1 and M2 phenotypes.

## Background

Traumatic brain injury (TBI) is responsible for 50% of trauma-related deaths in the United States and accounts for a substantial burden of disability in survivors [1,2]. To date, few effective treatments for acute TBI or its long-term sequelae have been identified [3]. The initial trauma to the brain triggers an inflammatory response, in which both resident microglia and peripheral macrophages play key roles [4].

Similar to the phenotypes of T cells in autoimmune and inflammatory diseases, different subsets of peripheral macrophages (inflammatory macrophages (M1) and anti-inflammatory macrophages (M2a and M2c)) have been identified in gliomas [5] and in breast and lung cancer [6,7], as well as after injury caused by acute peripheral nerve transection [8] and spinal cord injury [9]. The proinflammatory M1 phenotype is associated with tissue destruction [6,7,9,10], whereas the anti-inflammatory M2a and M2c phenotypes facilitate repair and regeneration in part by reducing inflammatory mediators [6,9,11].

Macrophages and microglia do not exist as immutable subsets; rather, they are sensitive to their host tissue microenvironments, with their phenotypes determined in part by their surroundings and the length of time after injury [9,12-15]. Macrophages can be differentiated *in vitro* into an M1 phenotype by lipopolysaccharide and interferon γ (IFN-γ) [16], resulting in classically activated macrophages that generate proinflammatory cytokines (for example, tumor necrosis factor α (TNFα)). The M2 subset results from activation of macrophages with interleukin 4 (IL-4) or IL-13 and can support reparative and regenerative processes [10,11,17,18].

The temporal relationship driving inflammatory and/or neurodegenerative processes versus reparative and/or neuroregenerative events following acute neurotrauma may be influenced by the ratio of M1 to M2 phenotypes [9-11,19]. Whether a similar schism exists in microglial and macrophagic phenotypes in the response to experimental TBI in the rat is currently unknown. We hypothesized that, after focal cortical contusion in the rat, larger numbers of M1 than M2 macrophages and microglia would be located within and around traumatic lesions.

The purpose of this study was to perform a time-course analysis of the macrophagic and microglial responses after TBI in the female rat. Tissue samples taken from brains postinjury were analyzed for expression of macrophagic and microglial markers by immunohistochemistry (IHC), flow cytometry and RNA and protein analysis. The results suggest that the postinjury environment results in a mixed population of microglia and macrophages rather than a milieu that is exclusively pro- or anti-inflammatory.

## Methods

### Animals

All studies were approved by the Institutional Animal Care and Use Committee at our institution. Experiments were performed according to the National Research Council’s *Guide for the Care and Use of Laboratory Animals*. Female Wistar rats (Charles River Laboratories, Wilmington, MA, USA) at an initial age of 8 weeks and weighing approximately 200 g (*N* = 72) were housed in pairs under 12-hour light–dark cycles in temperature-controlled conditions. Rats were given water and standard rodent laboratory chow *ad libitum*.

### Controlled cortical impact model

After the rats were anesthetized by nosecone administration of 2% to 3% isoflurane in 100% oxygen, the rat controlled cortical impact (CCI) model protocol [20-22] was carried out using an electromagnetic device (Impact One Stereotaxic Impactor (Leica Microsystems, Richmond, IL, USA) [23]. As previously described [24], a 6-mm craniotomy was followed by CCI with a 5-mm flat impactor tip over the left motor cortex (2.5 mm left lateral and 1.0 mm anterior from the bregma; velocity = 5 m/s, depth = 2.5 mm and dwell time = 100 ms). The same surgeon performed all CCI surgeries for a given cohort of rats.

### Cell isolation and flow cytometry

Control and post-CCI rats (*n* = 24) were killed with isoflurane and pentobarbital, then perfused with ice-cold, heparinized phosphate buffered saline (PBS). After each brain extraction, a 200-mg section centered over the injury site (or its equivalent in controls) was removed and placed into ice-cold Hanks’ balanced salt solution without calcium or magnesium. Tissues underwent enzymatic and mechanical dissociation with the MACS Neural Tissue Dissociation Kit (P) (Miltenyi Biotec, Bergisch Gladbach, Germany), followed by cell purification by centrifugation in an OptiPrep gradient medium (Axis-Shield, Oslo, Norway) to remove myelin and debris and to enrich the tissues for brain-derived macrophages and microglia as described previously [25].

After isolation, cells were fixed with BD Cytofix fixation buffer (BD Biosciences, San Jose, CA, USA) according to the manufacturer’s instructions. Cells (100,000 per reaction) were stained for CD40 (M1 marker), CD68 (generic macrophage marker) and CD163 (M2 marker) using antibodies against CD40 (eBioscience, San Diego, CA, USA) and against CD68 clone ED1 and CD163 (AbD Serotec, Kidlington, UK). Unstained cells and cells stained with appropriate isotype controls for each of the antibodies were used in control experiments. Stained cells and controls were analyzed on an Accuri C6 flow cytometer (BD Biosciences). A total of 30,000 events were counted in windows gated for the intersection of CD68 staining with CD40 and CD163.

### Immunohistochemistry

Rats (*n* = 24) were killed with isoflurane and pentobarbital and perfused with ice-cold, heparinized PBS followed by 4% paraformaldehyde. After extraction, perfused brains were placed into a sucrose gradient medium, transferred to optimal cutting temperature media and frozen in liquid nitrogen. Coronal plane cryosections (10 μm thick) were cut, mounted on Superfrost Plus glass slides (Thermo Scientific, Rockford, IL, USA) and stored at -20°C. Representative sections were examined by hematoxylin and eosin (H & E) staining using standard methods.

For IHC analysis, slides were washed with PBS, blocked in Super Block (ScyTek Laboratories, Logan, UT, USA) for 10 minutes at room temperature and incubated in primary antibody to detect the following: activated microglia and ionized calcium-binding adapter molecule 1 (Iba1) (Wako Chemicals, Richmond, VA, USA) at 1:200 dilution; or macrophages and CD68 (Abcam, Cambridge, MA, USA) at 1:200 dilution and CD86 (BD Biosciences) at 1:10 dilution; and CD163 (Hycult Biotech, Plymouth Meeting, PA, USA) at 1:10 dilution in 1× PBS, 0.3% Tween 20 and 1.0% bovine serum albumin at 4°C overnight. After being washed three times in PBS with 0.3% Tween 20, slices were incubated in secondary antibody (for CD68/CD86/CD163: goat F (ab’) secondary polyclonal antibody to mouse immunoglobulin G in H & L DyLight 649 (Abcam); for Iba-1: goat anti-rabbit F (ab’) Alexa Fluor 633 (H + L) (Life Technologies, Carlsbad, CA, USA)) at a dilution of 1:200 in 1× PBS with 0.3% Tween 20 and 1.0% bovine serum albumin at room temperature for 1 hour, rinsed three times in PBS with 0.3% Tween 20, dipped in distilled water and mounted in ProLong Gold Antifade reagent with 4′,6-diamidino-2-phenylindole (Invitrogen, Carlsbad, CA, USA). Slides were visualized with an Aperio ScanScope FL fluorescence microscope (Aperio/Leica Microsystems, Vista, CA, USA).

### Magnetic resonance imaging

After perfusion and paraformaldehyde fixation, a subset of the excised rat brains (*n* = 8) from the IHC cohort were immersed in susceptibility matching fluid (Fomblin PFPE lubricant; Solvay Solexis, Inc, West Deptford, NJ, USA) prior to IHC. Brains were imaged using a 7-T Bruker vertical bore magnet (Bruker BioSpin, Billerica, MA, USA) with a 10-mm inner diameter birdcage coil with a three-dimensional multiecho gradient echo sequence: repetition time (TR) = 200 ms, effective echo time (TE) = 17.5 ms (six echoes, 5 ms echo spacing), field of view = 3.0 × 1.5 × 1.25 cm^3^, 512 × 256 × 128 matrix, four averages and 30° flip angle as previously described [24].

### Immunofluorescent image analysis

For each animal, three brain slices per rat were selected from the center of the lesion and stained for a given antibody. Three regions of interest (ROIs) per tissue slice (20× magnification) were selected on the basis of consistent anatomical locations encompassing the medial, ventral and lateral aspects of the perilesion cortex. For each fluorophore, the same intensity thresholds were applied to quantify total fluorescent area using the Aperio ImageScope Positive Pixel Count Algorithm FL version 1 (Aperio/Leica Microsystems). Fluorescence quantification for each ROI was then averaged, divided by the total area of the region of interest (0.3979 mm^2^) and multiplied by 100 to calculate the percentage fluorescence. Immunofluorescence data were analyzed using Prism Mac version 6.0c software (GraphPad Software, San Diego, CA, USA) by two-way analysis of variance (ANOVA) with the Bonferroni correction using time and ROI location as variables, and by one-way ANOVA with a *post hoc* Tukey correction with time used as a variable.

### RNA and protein extraction

After rats (*n* = 24) were killed with isoflurane and pentobarbital, their brains were rapidly extracted and placed on ice. A 400-mg wedge of perilesion and lesion of injured cortex and striatum was removed, flash-frozen in liquid nitrogen and stored at -80°C. Prior to further extraction, frozen brain tissue was placed into prechilled Ambion RNA*later*-ICE reagent (Ambion/Life Technologies, Austin, TX, USA) and kept at -20°C for a minimum of 16 hours in accordance with the manufacturer’s instructions. Tissue was homogenized on ice in PARIS Cell Disruption Buffer (Ambion) supplemented with a protease inhibitor (Complete Protease Inhibitor Cocktail; Santa Cruz Biotechnology, Santa Cruz, CA, USA) with Omni Tip probes (Omni International, Kennesaw, GA, USA). RNA and protein fractions were processed from the same starting tissue samples using the Ambion PARIS kit per the manufacturer’s instructions and stored at -80°C. RNA concentrations and integrity were determined by analysis on Experion RNA StdSens chips (Bio-Rad Laboratories, Hercules, CA, USA). Protein concentrations were determined by bicinchoninic acid assay (Thermo Scientific).

### Quantitative RT-PCR

RNA was transcribed into cDNA using the SABiosciences RT^2^ First Strand Kit (QIAGEN, Valencia, CA, USA) according to the manufacturer’s instructions. Quantitative RT-PCR (qRT-PCR) was performed using the CFX96 Touch Real-Time PCR Detection System (Bio-Rad Laboratories) with SABiosciences RT^2^ SYBR Green qPCR Master Mix (QIAGEN). Pooled cDNA samples from each time point were first screened with the SABiosciences Rat Common Cytokines RT^2^ Profiler PCR Array or with specific primers for M1- and M2-associated markers (QIAGEN). The expression of genes of interest that showed significant up- or downregulation on this initial screen was then confirmed by running individual samples in triplicate for each time point on custom arrays using validated SABiosciences PCR primers for those rat genes (QIAGEN). Data were analyzed using SABiosciences RT^2^ Profiler PCR Array Data Analysis version 3.5 software and the SABiosciences RT^2^ Profiler PCR Array Data Analysis Template version 4.0 (QIAGEN).

### Cytokine and chemokine analysis

Protein samples at concentrations of 2 mg/ml were analyzed using the Bio-Plex Pro Rat Cytokine 23-plex Assay on a Bio-Plex 200 System and Bio-Plex Pro II Wash Station (Bio-Rad Laboratories) per the manufacturer’s instructions.

### Statistical analysis of RNA and protein data

Molecular data were analyzed by one-way ANOVA with a subsequent *post hoc* Tukey test using Prism Mac 6.0c software. A two-tailed *P* value <0.05 was considered to be significant. Errors are expressed as standard errors of the mean.

## Results

**Figure 1:**
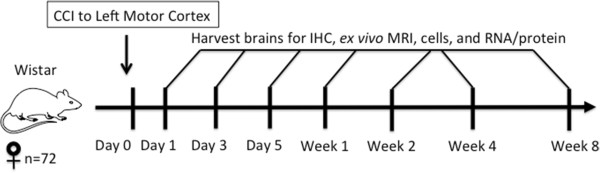
**Experimental design.** Female Wistar rats (*N* = 72) underwent controlled cortical impact (CCI) to the left motor cortex on day 0. Their brains were harvested (*n* = 9 per time point) for immunohistochemistry (IHC), *ex vivo* magnetic resonance imaging (MRI), flow cytometry and RNA/protein analysis over a time course ranging from 1 day to 8 weeks postinjury. Naïve control rats were subjected to the same protocol.

**Figure 2:**
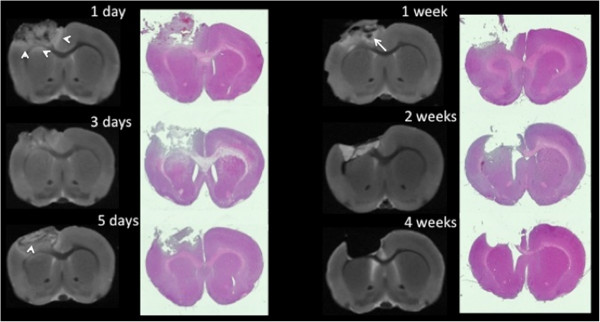
**Evolution of controlled cortical impact injury over time.***Ex vivo* magnetic resonance imaging (MRI) and corresponding hematoxylin and eosin stains demonstrate the controlled cortical impact (CCI) lesion’s extension from the cortex through the corpus callosum. By 4 weeks postinjury, the lesion had evolved into a cavity, with minimal further changes observed by 8 weeks (data not shown). Arrowheads on day 1 MRI scans indicate areas of edema. The arrow on the day 7 scan indicates an area of hemorrhage.

To determine the proinflammatory (M1) versus anti-inflammatory (M2) profiles of macrophages and microglia in the brain after CCI, we performed a serial time-course study (Figure 1). The rat brains were harvested for *ex vivo* magnetic resonance imaging (MRI), and tissues were processed for flow cytometry, IHC and RNA and protein analysis. In the first 24 hours after injury, *ex vivo* MRI and corresponding H & E–stained images demonstrated edema and tissue damage at the injury site (Figure 2). Over time, the damage evolved. Areas of hemorrhage developed by 1 week, which subsequently cleared over time, resulting in a cavity by 4 weeks postinjury that remained unchanged in size at 2 months after injury (data not shown).

**Figure 3:**
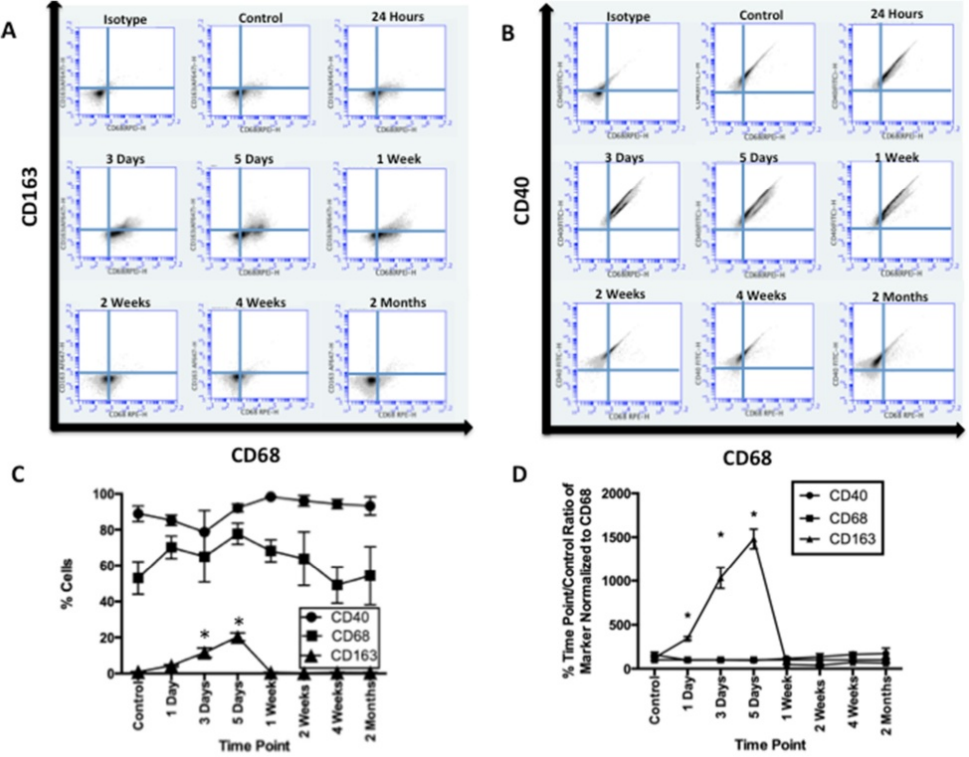
**Time course of inflammatory and anti-inflammatory macrophage markers in cells examined by flow cytometry.** Cells that stained for markers associated with macrophagic and microglial phenotypes were detected by flow cytometry of cells isolated from the lesion and/or perilesion area. **(A)** and **(B)** Comparison of cells stained with **(A)** general macrophage marker CD68 and anti-inflammatory macrophage (M2)-associated marker CD163 and **(B)** CD68 versus inflammatory macrophage (M1)-associated marker CD40 demonstrates a change in population occurring between 3 days and 1 week postinjury. **(C)** Statistically significant changes in the percentage of cells relative to controls that stained positive for the M2-associated marker CD163 were detected at 3 and 5 days postinjury (CD163: control = 0.9 ± 0.2%, 3 days = 12 ± 3%, 5 days = 20 ± 2%; *F* = 55.70). No significant differences were observed regarding the percentages of cells that stained for CD40 and CD68 over the time course. *P < 0.05. **(D)** Normalization of CD40 or CD163 to CD68 for flow cytometry data at each time point relative to controls allows better visualization of the degree of changes in M1 versus M2 ratios of macrophages. Data for CD163 are statistically significant at 1, 3 and 5 days postinjury by one-way analysis of variance (CD163: control = 120 ± 30%, 1 day = 350 ± 20%, 3 days = 1,000 ± 100%, 5 days = 1,500 ± 100%; *F* = 92.61). Error bars indicate standard errors of the mean. *P < 0.05.

Cells isolated from brain tissues for flow cytometry displayed characteristic macrophagic and microglial markers (Figure 3). Expression of the M2 marker CD163 was significantly different relative to baseline at 3 and 5 days postinjury (DPI) (CD163 expression as percentage of cells counted: control = 0.9 ± 0.2%, 3 DPI = 12 ± 3%, 5 DPI = 20 ± 2%; *F* = 55.70, *P* ≤ 0.0001 by one-way ANOVA). There were no significant differences in expression of the general macrophagic marker CD68 or the M1 marker CD40 in comparison to control values over the time course.

**Figure 4:**
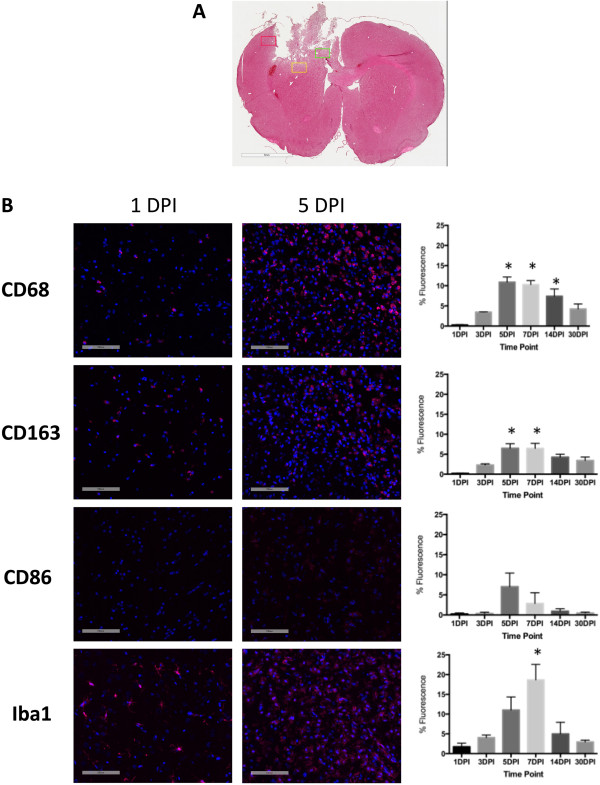
**Immunohistochemistry of macrophagic and microglial markers in brain tissue after traumatic brain injury over time.** Maximum immunofluorescence detection of markers of macrophagic and microglial polarization occurred by 5 days postinjury (DPI). **(A)** Representative anatomical localization of the regions of interest (ROIs; rectangles) quantified for the immunofluorescence of each marker. These ROIs were examined for the same anatomical slices from each animal. Scale bar = 3 mm. **(B)** Quantification of immunofluorescence in ROIs and representative immunofluorescent images at 1 DPI and 5 DPI. For a given time point, the column represents the average of all three ROIs for each slice, as shown in (A). Iba1, Ionized calcium-binding adapter molecule 1. Error bars indicate standard errors of the mean. *P < 0.05 for time point relative to 1 DPI. Scale bars = 100 μm.

Immunohistochemical staining for M1 and M2 markers on histological sections revealed a maximum generic macrophagic response (that is, CD68) at 5 DPI (Figure 4) (ROI: 1 DPI = 0.3 ± 0.1%, 5 DPI = 11 ± 2%; *F* = 15.11, *P* < 0.0001). Iba1 staining, characteristic of microglial activation, peaked at 1 week postinjury (ROI: 1 DPI = 1.8 ± 0.1%, 7 DPI = 19 ± 4%; *F* = 6.168, *P* = 0.0027). M2-associated CD163 staining was highest at 5 DPI (ROI (CD163): 1 DPI = 0.22 ± 0.09%, 5 DPI = 7 ± 1%; *F* = 5.764, *P* = 0.0043). Changes in M1-associated CD86 in comparison to 1 DPI were not significantly different by one-way ANOVA (ROI: 1 DPI = 0.3 ± 0.3%, 5 DPI = 7.0 ± 0.4%; *F* = 1.120, *P* = 0.4). Although a significant effect of time on staining for M1 and M2 markers was observed (two-way ANOVA for time for all markers: *F* > 4.5), no statistically significant differences were found among ROI locations by two-way ANOVA. Because of the lack of an effect of ROI location, the data are reported as the mean of all ROI locations for a given time point.

**Figure 5:**
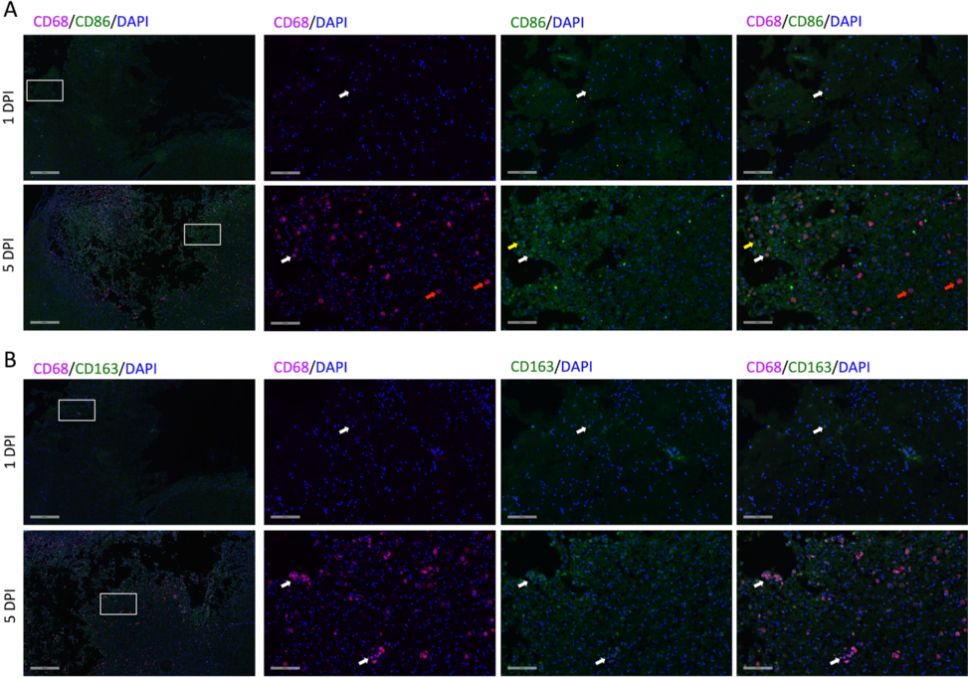
**Immunohistochemistry of macrophages double-labeled for CD68/CD86 or CD68/CD163.** CD68-positive macrophages express CD86 and CD163. **(A)** Representative immunofluorescent images double-labeled for CD68/CD86 and 4′,6-diamidino-2-phenylindole (DAPI) at 1 day postinjury (DPI) and 5 DPI. CD68-positive macrophages expressing the M1 marker CD86 were located predominantly in necrotic tissue (white arrows). Similarly, macrophages expressing only CD86 were located in necrotic tissue (yellow arrows). Macrophages expressing only CD68 were located in non-necrotic perilesional tissue (red arrows). **(B)** Representative immunofluorescent images double-labeled for CD68/CD163 and DAPI at 1 DPI and 5 DPI. All CD68-positive macrophages were positive for CD163 to varying degrees (white arrows). The leftmost images in each row display low-magnification (4X) images (scale bars = 500 μm), with the rectangles indicating the selected areas of higher magnification (20X) (scale bars = 100 μm) shown in the three accompanying images to the right.

Qualitative double-labeling of brain sections for CD68 and CD86 or for CD68 and CD163 demonstrated different localizations of M1 versus M2 macrophages (Figure 5). The CD86-positive CD68 macrophages were located predominantly in the necrotic area of the lesion, and the CD163-positive CD68 positive macrophages were found in the perilesion area.

**Figure 6:**
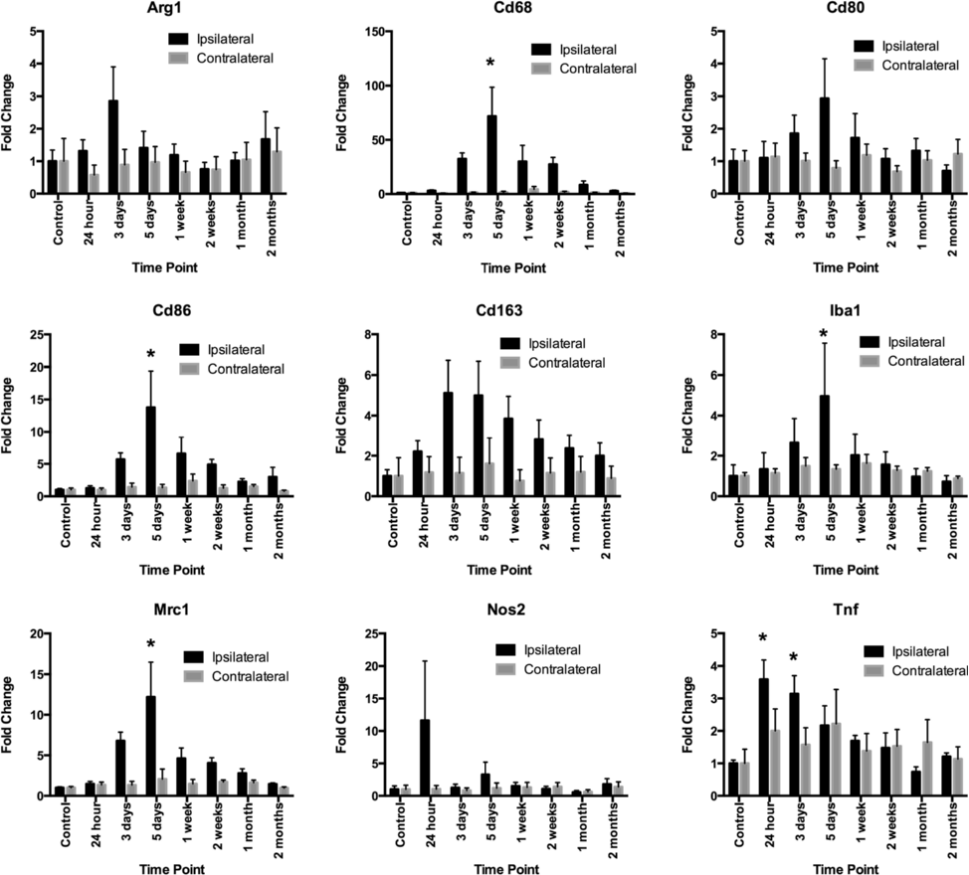
**Time course of inflammatory macrophage– and anti-inflammatory macrophage–associated gene expression in the brain after traumatic brain injury.** Changes in RNA expression of inflammatory macrophage (M1) and anti-inflammatory macrophage (M2) macrophagic and microglial markers peaked in ipsilateral brain tissue at 5 days postinjury (DPI) as assessed by quantitative RT-PCR. Statistically significant fold changes at 5 DPI relative to control and contralateral sides occurred for CD68, CD86, ionized calcium-binding adapter molecule 1 (Iba1) and mannose receptor, C type 1 (Mrc1). Changes in CD80 were significant when ipsilateral sides were compared to contralateral sides, but not in controls. In contrast, expression of tumor necrosis factor α (Tnf) was highest at 24 hours and 3 DPI, but there were no statistically significant fold changes in RNA expression of arginase 1 (Arg1), CD80, CD163 or nitric oxide synthase 2 (Nos2). Error bars indicate standard errors of the mean. *P < 0.05.

**Figure 7:**
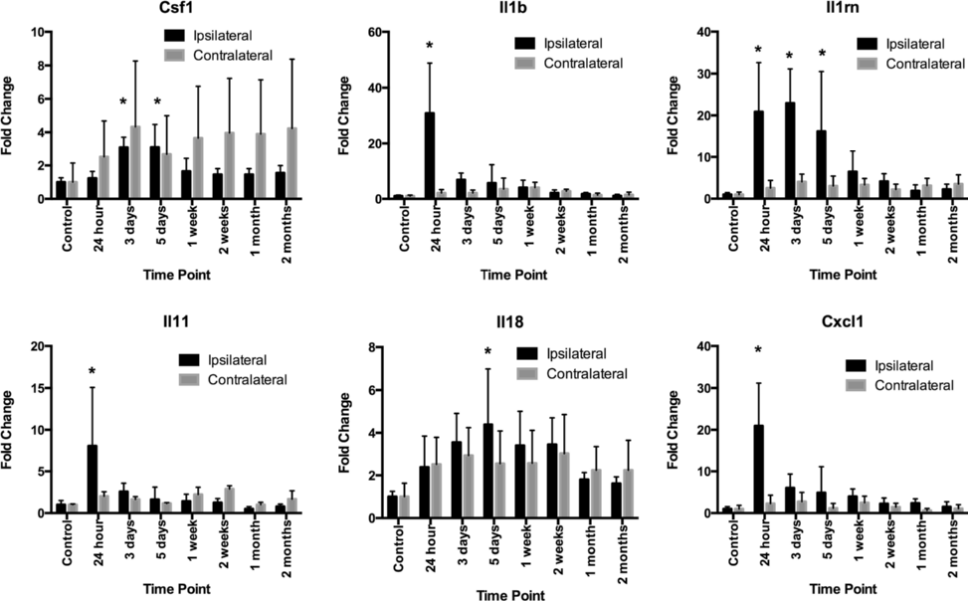
**Temporal changes in cytokine RNA expression in the brain after traumatic brain injury.** RNA expression of pro- and anti-inflammatory cytokines in ipsilateral brain changes in the first days to 8 weeks after injury. Initial fold changes were detected using pooled samples for each time point in a rat cytokine array. Fold changes greater than fourfold relative to baseline were subsequently verified in custom arrays by running individual samples in triplicate for each time point. Maximum fold changes were occurred for interleukin 1β (Il1β), IL-11 (Il11) and chemokine (C-X-C motif) ligand 1 (Cxcl1) at 24 hours postinjury. Maximum fold changes occurred for colony-stimulating factor 1 (Csf1) 3 to 5 days postinjury, IL-18 (Il18) peaked at 5 days postinjury and IL-1 receptor antagonist (IL-1rn) was elevated from 24 hours to 5 days postinjury. Error bars indicate standard errors of the mean. *P < 0.05.

RNA expression of markers CD68, CD86, Iba1 and mannose receptor, C type 1 (Mrc1) was highest relative to control and contralateral expression at 5 DPI (fold changes in CD68: control = 1 ± 0.2, 5 DPI = 72 ± 27 (*F* = 4.488, *P* = 0.0002); fold changes in CD86: control = 1 ± 0.2, 5 DPI = 14 ± 6 (*F* = 3.117, *P* = 0.0036); fold changes in Iba1: control = 1 ± 0.5, 5 DPI = 5 ± 3 (*F* = 3.720, *P* = 0.0011); fold changes in Mrc1: control = 1 ± 0.1, 5 DPI = 12 ± 4 (*F* = 3.976, *P* = 0.0006)) (Figure 6). TNFα increased significantly at days 1 and 3 after injury (fold changes in TNFα: control = 1 ± 0.1, 24 hours postinjury = 3.6 ± 0.6, 3 DPI = 3.1 ± 0.6 (*F* = 3.9, *P* = 0.0007)). No statistically significant differences were found in the expression of arginase 1 (Arg1), CD163 or nitric oxide synthase 2 (Nos2). RNA for chemokine (C-X-C motif) ligand 1 (CXCL1), cytokine IL-11 and IL-1β was maximal at 24 hours postinjury (Figure 7) (fold changes in CXCL1: control = 1 ± 0.3, 24 hours postinjury = 21 ± 6 (*F* = 11.10, *P* < 0.0001); fold changes in IL-11: control = 1 ± 0.3, 24 hours postinjury = 8 ± 4 (*F* = 3.221, *P* = 0.0031); fold changes in IL-1β: control = 1 ± 0.2, 24 hours postinjury = 31 ± 10 (*F* = 7.600, *P* < 0.0001)). Proinflammatory cytokine RNA for IL-18 peaked at 5 days (fold changes in IL-18: control = 1 ± 0.2, 5 DPI = 4 ± 2 (*F* = 2.688, *P* = 0.0103)). RNA expression of IL-1 receptor antagonist (IL-1ra), an anti-inflammatory cytokine, was elevated 15-fold from days 1 through 5, with a peak at 3 DPI (fold changes in IL-1ra: control = 1 ± 0.3, 3 DPI = 23 ± 5; *F* = 7.479, *P* < 0.0001).

**Figure 8:**
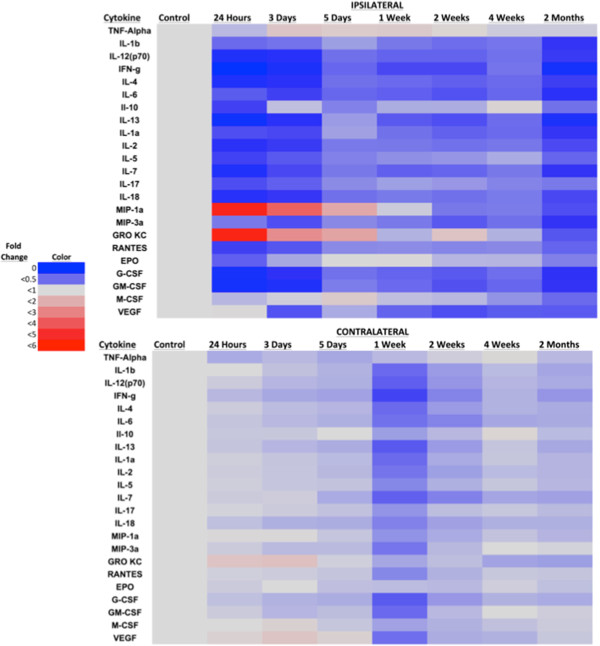
**Changes in cytokine and chemokine protein expression after traumatic brain injury.** The fold change protein expression of cytokines and growth factors assessed by multiplex assay decreased relative to expression in naïve controls in the combined lesion/perilesion area after injury, as summarized in the accompanying heat map. Chemokines macrophage inflammatory protein 1α (MIP-1a) and keratinocyte chemoattractant/growth-related oncogene (GRO KC) were the only proteins observed to have increased over the time course, peaking at 24 hours after injury, then decreasing. EPO, Erythropoietin; G-CSF, Granulocyte colony-stimulating factor; GM-CSF, Granulocyte-macrophage colony-stimulating factor; IFN-g, Interferon γ; IL, Interleukin; M-CSF, Macrophage colony-stimulating factor; RANTES, Regulated on activation, normal T cell expressed and secreted; TNF, Tumor necrosis factor; VEGF, Vascular endothelial growth factor. *P < 0.05.

The multiplex protein profiles of most cytokines from 24 hours through 2 months postinjury showed a statistically significant decrease in ipsilateral side cytokine levels relative to control and in the contralateral side at 24 hours (Figure 8 and Additional file 1: Figure S1). In contrast, macrophage inflammatory protein 1 α (MIP-1α) and keratinocyte chemoattractant/growth-related oncogene (KC/GRO, also known as CXCL1) increased significantly relative to control and the contralateral side at 24 hours postinjury (*P* < 0.05).

## Discussion

The following are the major findings of our serial time-course analysis after CCI in the female rat. (1) The maximum response of microglia and macrophages in the traumatically injured brain occurred within the first week after injury. (2) Markers indicative of pro- and anti-inflammatory activity are expressed within the first week after injury. (3) M1 and M2 markers return to near-baseline levels by 1 month after injury. To the best of our knowledge, our present study is the first in which the M1 and M2 responses of macrophages and microglia after CCI in female rats have been investigated. Comparisons of findings between published papers are complicated by animal strains used, animal sex, and methodological differences in studies using comparable as well as in different TBI models.

Cole *et al*. demonstrated that a sham craniotomy itself results in an inflammatory response at 1 DPI, with elevation of KC/GRO protein levels relative to naïve controls [26]. Greater changes were seen when the craniotomy was performed by using trephine versus drill. Therefore, for true comparison to baseline levels in the rat in the present study, naïve controls were used instead of craniotomy shams and all craniotomies were performed with drills. Consistent with reports of prior studies in which Sprague-Dawley male rats were used [27], we found activation of microglia in the hemisphere ipsilateral to injury in female Wistar rats after CCI.

By both flow cytometry and IHC, we observed a mixed pattern of M1 and M2 marker expression (Figures 3 and 4). M1 markers were present with the use of both techniques, but we did not find any statistically significant differences over the time course. M2 markers, however, peaked by 5 DPI and then decreased to control levels. Double-labeling experiments indicated that the majority of CD163+ cells localized to the perilesion area, whereas the CD86+ cells were located predominantly within the lesion itself (Figure 5). These observations support the idea that the M1 response is responsible for clearing dead tissue and that the M2 response is to preserve salvageable tissue in the perilesion area.

qRT-PCR analysis demonstrated a mixed pattern of expression for macrophage- and microglia-associated markers. Peak mRNA levels of the M1 markers CD80 and CD86, the M2 marker Mrc1 (CD206), the general macrophagic marker CD68 and the microglial marker Iba1 occurred at 5 DPI, with normalization occurring by 2 months after injury (Figure 6). qRT-PCR experiments carried out to study inflammation-related cytokines and chemokines showed a peak TNFα level at 24 hours postinjury, possibly indicative of its upregulation in resident microglia or in other cell types, such as astrocytes [28] or injured neurons [29], as peripheral macrophage infiltration is minimal at that time point. The upregulation of CXCL1, IL-1β and IL-11 RNA at 24 hours postinjury determined by qRT-PCR (Figure 7) may also be due to the presence of other cell types [30,31].

Analysis of selected cytokines and chemokines by multiplex assays demonstrated an almost universal decrease in cytokine and chemokine levels on the ipsilateral side relative to controls. The exceptions to these data were for proinflammatory chemokines MIP-1α and KC/GRO, which showed maximum increases at 24 hours, followed by decreases (Figure 8). The increases in these two chemokines are comparable to those reported by investigators in other studies [32,33].

In the present study, the population of brain macrophages and microglia after TBI shares characteristics of both pro- and anti-inflammatory markers. These results, in combination with those reported by Hsieh *et al*. [34] and others, suggest that these cells may fluctuate along a continuum between proinflammatory and anti-inflammatory responses, depending upon their environment after injury, rather than being at the extremes of the spectrum. Although some of these “classic” M1 and M2 markers change over the time course, others do not, suggesting that characteristics are more intermediate in these cell types after experimental TBI.

Researchers in two studies who used a mouse TBI model reported a mixed M1 and M2 response after CCI in male mice [34,35], with the M2 response peaking at 5 days and then rapidly decreasing by 1 week postinjury, with a return to baseline by 2 weeks. Zhang *et al*. [36] reported their use of the open-skull weight-drop model of TBI in Lewis rats (sex was not specified). They found parenchymal accumulation of M2 marker CD163 macrophages in the lesion between days 2 and 4 postinjury [36]; however, they obtained no data at time points beyond 96 hours. In our present study, CD163 expression peaked at 5 days. The slight temporal shifts between the diffuse weight-drop study [36] and our present study may be due in part to the different injury mechanisms and/or strain differences.

Researchers in several studies of mice and rats have investigated additional aspects of the neuroinflammatory response after experimental TBI. Lagraoui *et al*. [32] reported that CCI in male C56BL/6 mice induced a similar level of injury severity over a similar location (the motor cortex) compared to those in our study, and they examined the kinetics of the subsequent inflammatory response. Similarly to our present findings in rats, they found that RNA expression of CXCL1 and the proinflammatory cytokine IL-1β increased in expression early in the C56BL/6 mouse [32]. Peak expression of M1 marker CD86 in their study appeared closer to 7 days postinjury at the injury site compared to the 5 DPI peak observed in our present study. Their analysis of cytokine and chemokine expression by multiplex analysis post-CCI in the C56BL/6 mouse model showed similar upregulation of CXCL1 at 1 DPI. In contrast to the results of our present study, CCI in the mouse in their study resulted in significantly increased expression of IL-6, IL-1β, IL-10, IL-12p70 and IFNγ protein in their time course [32].

Kumar *et al*. [15] reported that, at 24 hours after CCI, substantial upregulation of RNA relative to sham controls for CD86, IL-1β, TNFα, Arg1 and inducible Nos2 in male mice ages 24 months and 3 months. Similar upregulation of TNFα and IL-1β RNA expression was found in the present study at 24 hours after CCI. Similar to young male mice in a previous study [15], the young female rats in our present study had little change in CD86 at 24 hours after CCI relative to controls. In contrast, by 5 DPI in female rats, we observed substantial fold changes in RNA expression by 5 DPI for CD68, CD86, Iba1 and Mrc1 (Figure 6). Examination of the RNA expression data from young male mice in a previous study [15] demonstrated few differences between young injured animals and controls at 24 hours postinjury. It is unknown whether older female rats would show upregulation of both pro- and anti-inflammatory markers at 24 hours postinjury, as was seen previously in older male mice [15].

At 24 hours post-CCI, previous authors reported that there was upregulation of KC/GRO in TBI relative to control male Sprague-Dawley rats [33]. In the present study, macrophage infiltration was not observed prior to 24 hours postinjury; therefore, we did not obtain data at earlier time points. The global decreases in protein expression in ipsilateral brain tissues relative to the contralateral side and to the control female rats observed were different from those reported in previous studies, which may reflect technical, strain or sex differences in response to TBI [33]. For example, Holmin *et al*. [37] used an open-skull (posterior) craniotomy weight-drop model in female Sprague-Dawley rats to investigate an inflammatory time course after injury. By *in situ* hybridization and immunohistochemistry, no expression of IL-1β, TNFα, IL-6 or IFNγ was detected within the first 2 days after injury. By days 4 to 6 postinjury, strong IL-1β, TNFα and IL-6 mRNA was detected by *in situ* hybridization [37], indicating a delayed response. In contrast, after a penetrating ballistic injury (PBI) in male Sprague-Dawley rats, upregulation of IL-1β, IL-6, IFNγ and TNFα by protein multiplex analysis was observed from 4 hours to 3 DPI in a previous study [38]. In the PBI model, neutrophil infiltration occurs at 24 hours postinjury, with a maximum microglial response by 72 hours [39], in contrast to our findings in our present study using the CCI model, in which the microglial response appeared to peak at 5 to 7 days. The differences in time course of cytokine expression in these different pathogenic TBI models [37-39] suggest considerable heterogeneity of inflammatory responses after TBI.

Multiple factors complicate the results obtained from the use of these different methods in our present study. Flow cytometry was performed on samples after enrichment using OptiPrep gradient medium to select for inflammatory cells, whereas neurons, oligodendrocytes and astrocytes were in nonanalyzed fractions of the gradient. In this cell population highly enriched for inflammatory cells, flow cytometry revealed that more than 50% of cells stained positive for CD68 and/or CD40 and less than 20% expressed CD163 at peak (Figure 3). In contrast, the brain slices used for IHC contained a heterogeneous cell population, diluting the concentration of immunological cells. Our comparisons were also complicated by the use of different M1 marker antibodies in the flow cytometry studies (CD40) and in IHC (CD86). A CD86 antibody for use in the rat was not available prior to the completion of the flow cytometry arm of this study. Initially, the M1 marker CD40 was also tested by IHC. Over the time course examined, however, the antibody demonstrated a high degree of background staining in nonmicroglial, nonmacrophagic cells in brain tissue (data not shown) in contrast to the enriched fraction of cells analyzed by flow cytometry, where minimal background staining was observed in the enriched cell population with antibody isotype controls (Figure 3B). The M1 marker CD86 subsequently used for IHC was more specific for macrophages and microglia in tissue sections.

For the flow cytometry arm of these investigations, the choice of species limited options for antibodies that could be used to further differentiate and gate cell populations. In mice, the antibody Ly6G can be used to exclude the neutrophil population and a CD45 antibody can distinguish macrophages (high CD45 expression) from microglia (low CD45 expression) [34,40]. Although a CD45 antibody is now available in rats, Ly6G is not. In addition, there are many more mouse antibodies available to distinguish M1 and M2 phenotypes than are currently available for rats (Table 1). A more refined cell sorting and gating strategy similar to that used by Bedi *et al*. [40] to separate macrophages (CD11b+, rat analogue to Ly6G - CD45+ (high expression)) from microglia (CD11b+, rat analogue to Ly6G - CD45+ (low expression)) would increase sensitivity for future flow cytometry studies to investigate M1 and M2 responses.

**Table 1:** **Antibody studies of M1 versus M2 phenotypes in neurotrauma**^**a**^

**Study**	**Injury model**	**Species**	**Sex**	**Methods**	**Cell type antibodies**	**M1 markers**	**M2 markers**
Bedi *et al*. [40]	CCI	Mouse	Male	FC	CD11b, CD45	CD16/CD32	CD206
Hsieh *et al*. [34]	CCI	Mouse	Male	FC, qRT-PCR on FC-sorted cells	CD11b, CD45, F4/F80, Ly6G	CD86, MHC II, YFP-IL-12p40	YFP-Arg1
Kigerl *et al*. [9]	SCI	Mouse	Not specified	IHC, qRT-PCR	None	CD16/CD32, CD86, iNOS, MHC II	Arg1, CD206
Kumar *et al*. [15]	CCI	Mouse	Male	IHC, qRT-PCR	CD11b, CD68, Iba1	MHC II	Ym1
Lagraoui *et al*. [32]	CCI	Mouse	Male	qRT-PCR, multiplex	None	None	None
Present study	CCI	Rat	Female	FC, IHC, qRT-PCR, multiplex	CD68, Iba1	CD40, CD86	CD163
Wang *et al*. [35]	CCI	Mouse	Male	IHC, qRT-PCR	Iba1	CD16/CD32	CD206
Zhang *et al*. [36]	CCI	Rat	Not specified	IHC	CD68	None	CD163

^a^*Arg1*, Arginine 1; *CCI*, Controlled cortical impact; *FC* = Flow cytometry; *Iba1*, Ionized calcium-binding adapter molecule 1; *IHC*, Immunohistochemistry; *IL*, Interleukin; *iNOS*, Inducible nitric oxide synthase; *MHC II*, Major histocompatibility complex class II; *qRT-PCR*, Quantitative RT-PCR; *SCI* = Spinal cord injury, *YFP* = Yellow fluorescent protein.

There is currently no antibody available in the rat that will exclusively identify microglia from macrophages. Although enrichment strategies can be attempted for flow cytometry studies, technical limitations currently make it difficult to distinguish between macrophages and microglia in a mixed population of cells, such as the brain tissue slices used for IHC or the brain tissue cores used for qRT-PCR in the present study. If an antibody that can reliably distinguish between macrophages and microglia is found, additional details related to M1 and M2 responses specifically in these cells in both the lesion and perilesion areas might be possible.

Factors underlying the observed differences in cytokine expression between the present study and others published in the literature include species (rat versus mouse), strain and sex differences as well as different methodologies. Methodological differences in the animal models (that is, depth of injury, CCI location, amount of hemorrhage at site of CCI), depending on species [24,32] and techniques (that is, tissue isolation, fresh versus flash frozen tissue for analysis) [33] used in tissue isolation and processing, may contribute to and explain differences between previously reported results and the results in the present study.

Genetic heterogeneity can also contribute to different outcomes after TBI [41,42] and can manifest as differences in immunological response. Nimer *et al*. [43] investigated genetic differences between two strains of inbred rats (Piebald Virol Glaxo (PVG) and Dark Agouti (DA)) that share similar major histocompatibility haplotypes but manifested differences in neuronal survival after TBI. Nimer *et al*. observed higher degrees of microglial and macrophagic activation, as well as greater activation of other immune cells, in the DA strain compared to the PVG strain in the CCI region. Genetic differences between strains are likely to be present to a greater degree in outbred strains, such as Wistar or Sprague-Dawley rats, commonly used in CCI studies.

In addition to genetic variability between strains and species, comparisons of results between studies can be confounded further by sex differences in the animals used. The response to neuroinflammation is regulated by estrogen levels via estrogen receptors [44]. Estrogen has been shown to have inhibitory effects on proinflammatory cytokines after diffuse TBI in rats [45]. Although the preinjury estrous stage has no correlation with outcome after experimental TBI in rats [46], female rats have better short-term behavioral outcomes after injury than males, suggesting that exposure to endogenous circulating sex steroids confers some neuroprotection against TBI. Whether TBI itself changes postinjury estrogen and progesterone levels in experimental animals is unknown; however, in clinical populations, both acute and chronic hypogonadotropic hypogonadism are known phenomena [47].

A differential neuroinflammatory response secondary to the short- and long-term effects of sex steroids may explain differences noted in studies of TBI in male versus female rodents. After experimental TBI, male rats have more brain edema [48] and brain injury [49] than cycling female rats, whereas ovariectomized female rats have levels of edema and injury similar to those of males. In gonadally intact female rats, estrogen and progesterone levels vary during the course of the 4- to 5-day estrous cycle [50,51]. After experimental cortical injury, pseudopregnant female rats, which have prolonged elevated progesterone levels, have less edema than normally cycling females [52]. Progesterone supplementation reduces edema and injury after experimental TBI in both female and male rats [48,53-57]. These preclinical studies suggest that exposure to sex steroid hormones, especially progesterone, can influence the response to TBI in experimental models.

## Conclusions

The present study demonstrates the complexity of the macrophagic and microglial responses after CCI in female Wistar rats. The brain microenvironment has elements of both pro- and anti-inflammatory responses within the first week after traumatic injury, rather than one that is clearly skewed more toward an M1 or M2 phenotype. A spectrum of macrophagic and microglial phenotypes exists after CCI, likely reflecting a complex inflammatory response in which the cells may adjust their functions based upon the posttraumatic milieu. This mixed pattern has relevance to translation of results to clinical populations. Future studies are needed to address the contributions of strain, species, sex and age differences to the neuroinflammatory response after TBI.

## Abbreviations

ANOVA: Analysis of variance; Arg1: Arginase 1; CCI: Controlled cortical impact; CXCL1: Chemokine (C-X-C motif) ligand 1 (also known as Growth-related oncogene); DA: Dark agouti rat strain; DAPI: 4′,6-diamidino-2-phenylindole; DPI: Days postinjury; ELISA: Enzyme-linked immunosorbent assay; H & E: Hematoxylin and eosin; Iba1: Ionized calcium-binding adapter molecule 1; IHC: Immunohistochemistry; IL: Interleukin; KC/GRO: Keratinocyte chemoattractant/growth-related oncogene (also known as chemokine (C-X-C motif) ligand 1); MIP-1α: Macrophage inflammatory protein 1α; Mrc1: Mannose receptor, C type 1; MRI: Magnetic resonance imaging; Nos2: Nitric oxide synthase 2; PBS: Phosphate-buffered saline; PVG: Piebald Virol Glaxo rat strain; qRT-PCR: Quantitative real-time polymerase chain reaction; ROI: Region of interest; SCI: Spinal cord injury; TBI: Traumatic brain injury; TNFα: Tumor necrosis factor α.

## Competing interests

The authors declare that they have no competing interests.

## Authors’ contributions

LCT participated in the design and coordination of the study, performed animal experimental procedures, conducted flow cytometry and molecular analyses and drafted the manuscript. JL conducted immunohistochemical analyses. LJ performed animal experimental procedures and conducted *ex vivo* MRI and immunohistochemical analyses. DDD performed animal experimental procedures, designed the immunohistochemistry protocols and assisted in data analysis and interpretation. MDB designed the *ex vivo* MRI protocols. JAF conceived of the study, participated in its design and helped draft the manuscript. All authors read and approved the final manuscript.

## Supplementary Material

Additional file 1: Figure S1 Protein concentrations of cytokines and chemokines after traumatic brain injury. After traumatic brain injury (TBI), the protein concentrations of most cytokines, chemokines and growth factors decreased on the side ipsilateral to TBI as assessed by Bio-Plex multiplex system panel (Bio-Rad Laboratories, Hercules, CA, USA). EPO, Erythropoietin; G-CSF, Granulocyte colony-stimulating factor; GM-CSF, Granulocyte-macrophage colony-stimulating factor; GRO KC, Growth-related oncogene (also known as chemokine (C-X-C motif) ligand 1); IFN, Interferon; IL, Interleukin; M-CSF, Macrophage colony-stimulating factor; MIP, Macrophage inflammatory protein; RANTES, Regulated on activation, normal T cell expressed and secreted; TNF, Tumor necrosis factor; VEGF, Vascular endothelial growth factor. Error bars indicate standard errors of the mean. *P < 0.05 for the indicated ipsilateral side time point relative to the control value. **P < 0.05 for the ipsilateral side versus the contralateral side. Click here for file
