# Supplementary figures and images for: Macrophagic and microglial responses after focal traumatic brain injury in the female rat
Source: J Neuroinflammation. 2014 Apr 24;11:82. doi: 10.1186/1742-2094-11-82 (PMC4022366; doi:10.1186/1742-2094-11-82)

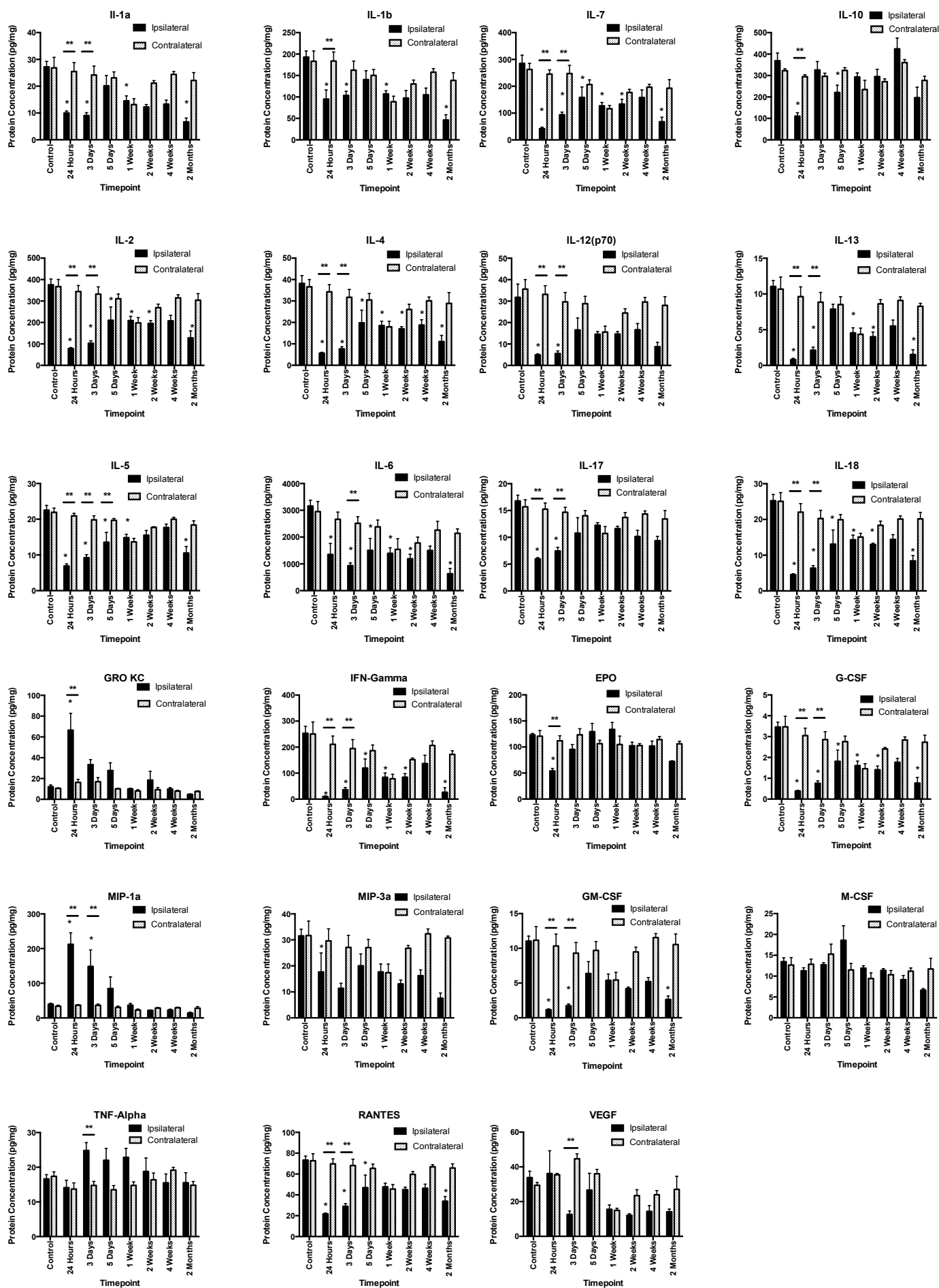

Supplement: Additional file 1: Figure S1 — Protein concentrations of cytokines and chemokines after traumatic brain injury. After traumatic brain injury (TBI), the protein concentrations of most cytokines, chemokines and growth factors decreased on the side ipsilateral to TBI as assessed by Bio-Plex multiplex system panel (Bio-Rad Laboratories, Hercules, CA, USA). EPO, Erythropoietin; G-CSF, Granulocyte colony-stimulating factor; GM-CSF, Granulocyte-macrophage colony-stimulating factor; GRO KC, Growth-related oncogene (also known as chemokine (C-X-C motif) ligand 1); IFN, Interferon; IL, Interleukin; M-CSF, Macrophage colony-stimulating factor; MIP, Macrophage inflammatory protein; RANTES, Regulated on activation, normal T cell expressed and secreted; TNF, Tumor necrosis factor; VEGF, Vascular endothelial growth factor. Error bars indicate standard errors of the mean. *P < 0.05 for the indicated ipsilateral side time point relative to the control value. **P < 0.05 for the ipsilateral side versus the contralateral side. [file 1742-2094-11-82-S1.pdf]
